# Effects of the microbial community on the formation of volatile compounds and biogenic amines during the traditional brewing of *Hongqu* rice wine

**DOI:** 10.1016/j.crfs.2022.08.020

**Published:** 2022-09-06

**Authors:** Gui-Mei Chen, Wen-Long Li, Shan-Gong Tong, Yun-Tao Qiu, Jin-Zhi Han, Xu-Cong Lv, Lian-Zhong Ai, Jin-Yuan Sun, Bao-Guo Sun, Li Ni

**Affiliations:** a College of Chemical Engineering, Fuzhou University, Fuzhou, Fujian, 350108, PR China; b Institute of Food Science and Technology, College of Biological Science and Engineering, Fuzhou University, Fuzhou, Fujian, 350108, PR China; c Food Nutrition and Health Research Center, School of Advanced Manufacturing, Fuzhou University, Jinjiang, Fujian, 362200, PR China; d Beijing Laboratory for Food Quality and Safety, Beijing Technology and Business University, Beijing, 100048, PR China; e School of Medical Instruments and Food Engineering, University of Shanghai for Science and Technology, Shanghai, 200093, PR China; f Fujian Huizelong Alcohol Co., Ltd, Pingnan County, Ningde, Fujian, 352303, PR China

**Keywords:** *Hongqu* rice wine, Microbial community, Volatile flavor components, Biogenic amines, Bioinformatical analysis

## Abstract

As a typical representative of Chinese rice wine (*Huangjiu*), *Hongqu* rice wine is famous for its red color, mellow taste and strong fragrance. However, due to the open brewing environment and traditional fermentation technology, there are some safety risks in traditional brewed *Hongqu* rice wine, such as a certain amount of biogenic amines. In this study, the dynamic changes and the differences of microbial communities and volatile flavor components between two types of *Hongqu* rice wine with high and low biogenic amine contents (LBAW and HBAW) during the traditional brewing were systematically investigated. The results showed that the total biogenic amine contents in LBAW and HBAW were 20.91 and 69.06 mg/L, respectively. The contents of putrescine, cadaverine, spermine and spermidine in HBAW were significantly higher than those in LBAW, and it was noteworthy that spermine content in HBAW was 17.62 mg/L, which was not detected in LBAW. In addition, the volatile flavor characteristics of the two kinds of *Hongqu* rice wine were obviously different. The contents of acetophenone, n-butyl butanoate and benzothiazole were obviously higher in HBAW, while the contents of isoamyl acetate, ethyl lactate, ethyl caprate and phenylethyl alcohol were significantly higher in LBAW. High-throughput sequencing of 16S/ITS amplicon revealed that *Weissella*, *Kosakonia*, *Pantoea*, *Monascus*, *Saccharomyces* and *Millerozyma* were the predominant microbial genera during the traditional brewing of HBAW, while *Weissella*, *Kosakonia*, *Monascus*, *Saccharomyces* and *Issatchenkia* were the predominant microbial genera during the traditional brewing of LBAW. Correlation analysis revealed that biogenic amines were significantly negatively correlated with unclassified_o_*Saccharomycetales*, *Cyberlindnera*, *Zygoascus*, *Aspergillus* and *Acinetobacter*, but positively correlated with *Lactobacillus*, *Pediococcus*, *Millerozyma* and *Apiotrichum*. In addition, we also found that *Lactobacillus*, *Pediococcus* and *Saccharomyces* were significantly positively correlated with most of the volatile flavor components, while *Candida*, *Trichosporon* and *Monascus* were significantly negatively correlated with most of the volatile flavor components. In addition, bioinformatical analysis based on PICRUSt demonstrated that the key enzymes for biogenic amine biosynthesis were more abundant in the microbial community of HBAW than LBAW. These findings demonstrate that the formations of volatile flavor and biogenic amines in *Hongqu* rice wine are influenced by microbial community during the fermentation. This work facilitates scientific understanding of the formation mechanism of biogenic amines, and may be useful to develop effective strategies to improve the quality of *Hongqu* rice wine.

## Highlights

• The total contents of biogenic amines in LBAW and HBAW were 20.91 and 69.06 mg/L, respectively.
• The volatile flavor characteristics of LBAW and HBAW was quite different.
• Biogenic amines were significantly positively correlated with *Lactobacillus*, *Pediococcus*, *Millerozyma* and *Apiotrichum*, *etc.*
• The key enzymes for the biosynthesis of biogenic amines were more abundant in the microbial community of HBAW than LBAW.

## Graphical abstract

**Figure:**
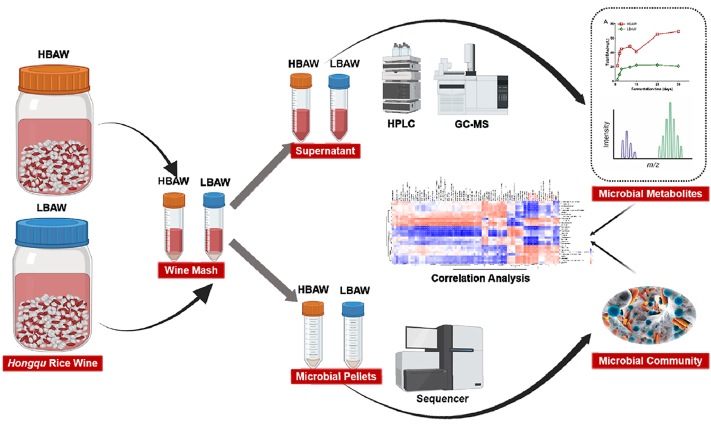

## 1 Introduction

Yellow rice wine (also known as *Huangjiu*), known as the national wine of China, has been favored by Southeast Asian consumers in recent years, making the rapid increasing of its production and sales. As a typical representative of Chinese rice wine, *Hongqu* rice wine possesses bright red color, subtle sweet taste, typical mellow flavor and health functions due to the addition of *Hongqu* (red mold rice). As a starter for *Hongqu* rice wine, *Hongqu* is made from steamed rice by solid-state fermentation with the inoculation of starter cultures (*Qumu*, *Monascus* as the predominant microbe) (Liu et al., 2018; Zhou et al., 2021). Similar to other traditional fermented foods, *Hongqu* is usually prepared in a non-sterile, open fermentation environment. Thus, a wide variety of microorganisms are presented in *Hongqu*, and the quality of starter varies from region to region, which leads to the uncontrollability of the fermentation process and product quality of *Hongqu* rice wine. The traditional brewing of *Hongqu* rice wine is a process of “co-fermentation of various microorganisms”, and its quality are closely related to the unique microbial community characteristics and metabolic functions in the whole brewing process. *Hongqu* rice wine brewing involves the joint participation of various molds, yeasts and bacteria. The composition of microbial community in the brewing process are closely related to the flavor and quality of *Hongqu* rice wine. However, there are also some undesirable microorganisms, potential pathogens and even spoilage bacteria that can produce some harmful metabolites, such as ethyl carbamate and biogenic amines, in the traditional brewing process of *Hongqu* rice wine (Xia et al., 2018; Zhou et al., 2017).

Biogenic amines (BAs), a general term of low-molecular-weight nitrogenous organic compounds, are usually generated by through enzymatic decarboxylation of free amino acids or amination and transamination of aldehydes and ketones by microbial metabolism (Latorre-Moratalla et al., 2010). Excessive intake of foods containing BAs may cause adverse physiological reactions, such as increased blood pressure, headaches, vomiting, heart palpitations, heart failure and digestive disorders (Maintz and Novak, 2007; Benkerroum, 2016; Pegg, 2013; Sun et al., 2020; Schirone et al., 2022). The most common BAs in fermented foods include putrescine, histamine, tyramine, tryptamine, phenylethylamine, cadaverine, spermine and spermidine. BAs can be oxidized to aldehydes, such as histamine to imidazole acetaldehyde, tyramine to hydroxyl phenylacetaldehyde, *etc.* This oxidation reaction is mediated by amine oxidase, an enzyme widely present in mammals and microorganisms that metabolizes BAs to a low physiological level. Unfortunately, low concentration ethanol intake may significantly inhibit the activity of amine oxidase in BAs catabolism (Anli and Bayram, 2009; Garcia-Ruiz et al., 2011). Therefore, the accumulation of BAs in alcoholic fermented foods is a major health concern due to their potential toxicity. However, due to the different toxicity thresholds and the cumulative toxicity of different kinds of BAs, it is difficult to establish BAs limit standards for alcohol fermented foods. The limit of BAs in rice wine has not been clearly stipulated at home and abroad.

The formation of biogenic amines during the traditional brewing of rice wine is mainly attributed to the presence of decarboxylase-producing microorganisms (Ordóñez et al., 2016; Luo et al., 2020). In the process of rice wine brewing, the protease and carboxypeptidase secreted by Aspergillus and Monascus act on proteins in glutinous rice to produce amino acids or oligopeptides, which are further decarboxylated into the corresponding BAs. According to a previous report, the content of amino acids in the traditional brewing process of industrial semi-dry yellow rice wine is 365.53–3243.35 mg/L (Xia et al., 2016), which provided abundant precursor substances for the formation of BAs (Zhong et al., 2012). The average content of BAs in rice wine (∼115 mg/L) is much higher than that in liquor (∼0.65 mg/L), grape wine (∼11.24 mg/L) and beer (∼4.79 mg/L) (Lu et al., 2007; Zhong et al., 2012). Previous study found that BAs are the main intoxicating compounds in rice wine, and drinking rice wine with a higher content of BAs is more likely to cause negative physiological effects than drinking red wine (Sun et al., 2020). Although the composition and content of BAs in different types of rice wine are different, the main biogenic amines in rice wine are putrescine, tyramine, cadaverine, spermine and histamine (Zhong et al., 2012; Zhong et al., 2012). It well known that the formation of BAs in rice wine is closely related to the metabolic activity of decarboxylase-producing microorganisms for amino acids. Current studies generally believe that lactic acid bacteria are the main biogenic amines producers in fermented foods (Ruiz-Capillas and Herrero, 2019). *Oenococcus*, *Lactobacillus*, *Leuconostoc*, *Photobacter* and *Pseudomonas* are the main producers of BAs in wine production (Coton et al., 2010; Moreno-Arribas et al., 2003; Liu et al., 2021). Although previous studies have reported the composition of microbial flora and its dynamic changes during the traditional brewing process of *Hongqu* rice wine (Liu et al., 2020; Huang et al., 2019; Huang et al., 2018), the relationship between the microbial community and the formation of BAs has not yet been revealed.

The current study aimed to explore the effects of the microbial community on the formation of volatile compounds and BAs during the traditional fermentation of *Hongqu* rice wine. High-throughput sequencing of amplicon was applied to detect the differences of microbial community of the two types of *Hongqu* rice wine with high and low BAs contents (LBAW and HBAW) during the traditional brewing. Besides, the volatile flavor compounds and BAs produced during the traditional brewing were detected through gas chromatography-mass spectrometry (GC-MS) and high performance liquid chromatography (HPLC), respectively. Furthermore, the potential correlations between the dominant microbial genera and BAs, as well as volatile flavor compounds, were visualized through correlation heatmaps. Finally, Phylogenetic Investigation of Communities by Reconstruction of Unobserved States (PICRUSt) analysis was used to revealed the key enzymes for the biosynthesis of BAs. This research will provide a theoretical basis for the green and safe production of *Hongqu* rice wine in industrial manufacturing scale.

## 2 Materials and methods

### 2.1 Samples collection and pretreatment

Two distinct fermentation starters (*Koji*) for *Hongqu* rice wine brewing were purchased from Fujian Huizelong Alcohol Co., Ltd (Pingnan, Fujian, China) and Fujian Baishuiyang Alcohol Co., Ltd (Pingnan, Fujian, China), and used to produce *Hongqu* rice wine through traditional brewing techniques. The mash samples were collected form the fermentation process of the two types of *Hongqu* rice wine, namely LBAW (fermented with the *Koji* from Fujian Huizelong Alcohol Co., Ltd) and HBAW (fermented with the *Koji* from Fujian Baishuiyang Alcohol Co., Ltd). Moreover, the preparation of *Hongqu* rice wine was conducted according to the previously described method (Zhao et al., 2022). In total, seven time points with triplicate fermentation samples for the analyses of oenological parameters, volatile profiles and microbial community were collected (day 1, day 2, day 3, day 7, day 10, day 20 and day 30). Mash samples of *Hongqu* rice wine during the traditional brewing were collected and sealed in vials, then stored at −80 °C for further testing. Mash sample (10 g) was taken and put into 90 mL sterile saline and filtered with gauze after shaking with a vortex shaker for 30 s, the supernatant was then collected after being centrifuged at 8000 r/min, for 10 min, and finally stored at −20 °C for the oenological parameters analysis. Meanwhile, the obtained microbial pellets were stored at −80 °C for high-throughput sequencing analysis.

### 2.2 Detections of the oenological parameters

#### 2.2.1 Determination of total acids

The total acid content was determined using the method described by National Standards of China (GB/T 5517–2010) and a previous study (Yang et al., 2018) with minor modification. 5 mL of supernatant and 25 mL of CO2-free water were added to a 100 mL beaker, and then titrated with sodium hydroxide standard titration solution, while pH 8.20 is regarded as the end point of titration.

#### 2.2.2 Determination of reducing sugar

The determination of reducing sugar was conducted on the basis of 3,5-dinitrosalicylic acid (DNS) method (Miller, 1959). Briefly, 1 mL sample was mixed with 2.0 mL DNS reagent and bathed in boiling water for 5min. After that, it was cooled quickly with flowing water, supplemented with distilled water to 25 mL and shaken well. Absorbance was measured at 540 nm.

#### 2.2.3 Determination of alcohol

The alcohol content was determined by gas chromatography (GC, 7890A, Agilent, USA) with FID (flame ionization detector) and HP-INNOWAX column (30.0 m × 0.25 mm × 0.25 μm, Agilent, USA). The injection volume was 1 μL. The oven temperature was programmed at 40 °C for 5 min, increased at 20 °C/min to 220 °C, and held at 220 °C for 5 min. The splitting ratio of injection was set at 10:1. The flow rate of H_2_ was 30 mL/min. The flow rate of air was 400 mL/min. The flow rate of tail blow (N_2_) was set at 25 mL/min.

#### 2.2.4 Determination of amino acid nitrogen

The determination of amino acid nitrogen in mash samples of *Hongqu* rice wine was conducted according to a previous study (Zhao et al., 2020).

### 2.3 Determination of BAs

The content of BAs during the traditional brewing of *Hongqu* rice wine was determined using the method described by Pineda et al. (2012) with some modifications. 1 mL samples and 0.05 g polyvinylpyrrolidone (PVP) were added into a 2 mL centrifuge tube and mixed well, and oscillated at constant temperature (25 °C and 500 rpm) for 15 min, then subsequently filtered with Whatman Grade No. 4 qualitative filter paper. Next, 100 μL filtrate was pipetted into a 2-mL centrifuge tube, and 400 μL sodium carbonate-sodium bicarbonate buffer and 300 μL acetone and 200 μL danyl sulfonyl chloride solution were added. After vortex mixing for 30 s, the solution was bathed in water at 60 °C for 60 min. After filtration with a 0.22 μm membrane, the samples were placed in 2 mL liquid injection vials for high-performance liquid chromatography (HPLC, Agilent 1100) with a ZORBAX SB-C18 column (4.6 mm × 250 mm, 5 μm). The mobile phases of liquid chromatography were deionized water (A) and acetonitrile (B), and the elution procedures were as follows: 0–6.0 min 35% B, 6.01–16.0 min 35%–55% B, 16.01–24.0 min 55%–60% B, 24.01–48.0 min 60%–90% B, 48.01–53.0 min 90%–35% B.

### 2.4 Volatile profiles analyses

The profile of volatile flavor substances was mainly determined using head-space solid-phase microextraction (HS-SPME) combined with GC-MS instrument (Agilent 7890-B/5977A). The sample treatment and instrument operation were performed according to the (Liu et al., 2020; Huang et al., 2019) with some modifications. Briefly, supernatant (6 mL) was added into 15-mL headspace glass vials with 2 g of sodium chloride and 10 μL internal standard of 2-octanol (10 mg/L). The 50/30 μm divinylbenzene/carboxen/poly (dimethylsiloxane) (DVB/CAR/PDMS) fiber (2 cm, Supelco, Bellefonte, PA, USA) in a solid-phase microextraction (SPME) device (Supelco, Bellefonte, PA) was inserted into the vial, then the vial was equilibrated at 60 °C for 10 min and extracted for 45 min at 60 °C under stirring to extract and adsorb volatile compounds. The volatile compounds were then desorbed at 250 °C into the GC inlet for 5 min with splitless mode. The GC-MS equipped with HP-INNOWAX capillary column (30.0 m × 0.25 mm × 0.25 mm, Agilent Technology, USA) was used to measure the volatile compounds of each sample. The oven temperature was programmed at 40 °C for 5 min, increased at 5 °C/min to 120 °C, ramped to 240 °C at a rate of 10 °C/min and held for 5 min at this final temperature. The post operating temperature was 250 °C, held for 5min. The flow rate of carrier gas (helium) was 1 mL/min. The MS interface temperature was set at 280 °C, and the MS quadrupole was 150 °C. The electron ionization (EI) mass spectra mode was used at 70 eV ionization energy. And the temperature of the ion source was 230 °C. The chromatogram was recorded by monitoring the total ion currents in the m/z range of 35–450. Compare with the database of National Institute of Standards and Technology (NIST), record and identify volatile peaks. The analysis of the sample was made in triplicate.

### 2.5 DNA extraction and sequencing analysis

Total DNA was extracted from the precipitate according to the protocol of rapid DNA extraction kit (MN NucleoSpin 96 Soil, Germany). The extracted total DNA was used as a template to amplified bacterial 16S rDNA V3–V4 region by 338F (5′-ACTCCTACGGGAGGCAGCAG-3′) and 806-R (5′-GGACTACHVGGGTWTCTAAT-3′) primers (Xu et al., 2016), fungal internal transcribed spacers (ITS) by ITS5-1737-F (5′-GGAAGTAAAAGTCGTAACAAGG-3′) and ITS2-2043-R (5′-GCTGCGTTCTTCATCGATGC-3′) primers (Zhang et al., 2016). The PCR products were used for subsequent DNA library construction, and the two libraries were sequenced using Illumina HiSeq 2500 instrument (Illumina, CA, USA). After the sequencing data were spliced and filtered according to the overlapping relationship, the quality of the sequences was controlled and screened. Raw sequencing reads were quality-filtered and analyzed using FLASH software (v1.2.7) and QIIME software (v1.8.0). Operational taxonomic unit (OTU) clustering analysis was performed at sequence similarity of 97% based on the UPARSE algorithm using USEARCH software (Version 7.1). The bacterial OTU sequences were annotated using the SILVA/16S rDNA database (Quast et al., 2012) by a QIIME-based wrapper of the RDP-classifier (v.2.2) (Cole et al., 2009). The OTU sequences of fungal ITS were clustered using the UNITE database (Kõljalg et al., 2013) by USEARCH (version 5.2.236) and aligned by the BLAST algorithm.

### 2.6 Statistical analysis

Volatile component composition and functional gene abundance were visualized through heatmap and bubble matrix packages in R software, respectively. Principal component analysis (PCA) of volatile components was performed using SIMCA-14.1 software to evaluate the variation trend of volatile flavor characteristics of *Hongqu* rice wine during traditional brewing process. Subsequently, the Spearman correlation coefficients between the volatile components/biogenic amines and the microbial taxa during the traditional brewing of *Hongqu* rice wine were calculated and visualized through Psych, Reshape2 and Pheatmap packages in R software.

## 3 Results and discussion

### 3.1 The dynamics of physicochemical parameters

**Fig. 1:**
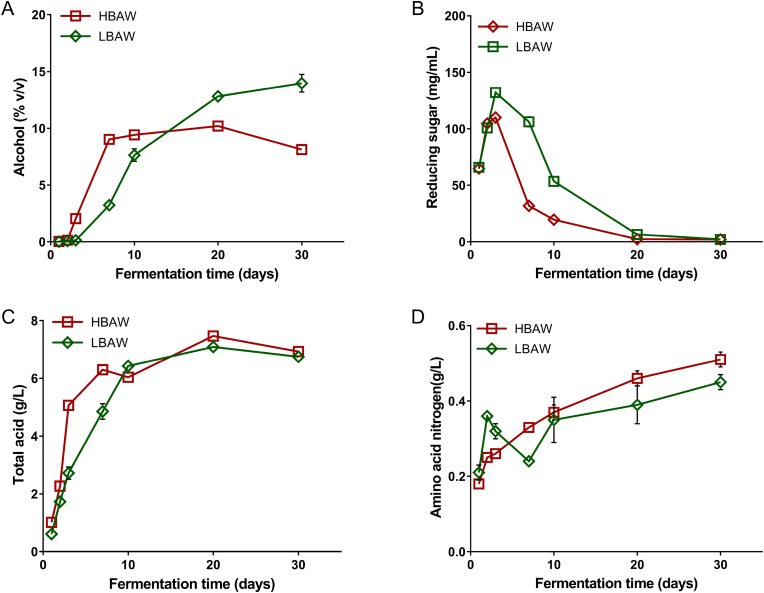
Dynamic changes of ethanol (A), total acids (B) and reducing sugar (C) during the traditional brewing of two types of *Hongqu* rice wine (LBAW and HBAW).

Physicochemical parameters including alcohol, reducing sugar, total acids and amino acid nitrogen are considered as the important parameters to measure the flavor quality of *Hongqu* rice wine, which can reflect the metabolic activity of microorganisms in the fermentation process to some extent (Zhao et al., 2020). As showed in Fig. 1A, we observed the dynamic changes of the above physicochemical parameters in the brewing process of HBAW and LBAW. The alcohol content of HBAW and LBAW showed an increasing trend during the whole fermentation process, especially in the early and middle stages of fermentation. However, in the later stage of fermentation, the alcohol level remained stable and almost reached the plateau stage, This may be due to the consumption of nutrients and the stress of ethanol and acid, which greatly restricted the growth and metabolism of yeast (Longo et al., 2021). Notably, the alcohol content of HBAW was significantly lower than that of LBAW at the end of fermentation. In addition, in the early stage of fermentation, the reducing sugar content of the two kinds of *Hongqu* rice wine (HBAW and LBAW) increased sharply, reaching the peak of 110.05 mg/mL (HBAW) and 132.17 mg/mL (LBAW), respectively, and then showed a sharp downward trend (Fig. 1B). At the initial stage of fermentation, starch is hydrolyzed into small molecules of reducing sugars including glucose and maltose under the action of α-amylase and glucoamylase (Deng et al., 2020). Subsequently, the reducing sugar is consumed by alcohol-producing yeasts, molds, lactic acid bacteria to produce metabolites such as alcohol and lactic acid, and so on, resulting in a decreasing trend of reducing sugar content. In addition, the total acids content of the two kinds of *Hongqu* rice wine (HBAW and LBAW) also increased dramatically in the early stage of fermentation, and then increased slowly and tended to be stable in the subsequent fermentation process. It is worth noting that there was no significant difference in the total acids level between HBAW and LBAW (Fig. 1C). In a previous study, it was demonstrated that the content of organic acids in rice wine was positively correlated with the abundance of lactic acid bacteria during brewing (Wang et al., 2014). Therefore, lactic acid bacteria are widely considered as the main contributors of total acid in rice wine. The amino acid nitrogen contents of HBAW and LBAW increased gradually (Fig. 1D). We also observed that the content of amino acid nitrogen decreased obviously on the 7th day of LBAW fermentation, which may be due to the absorption and utilization of amino acids by some microorganisms. In general, the saccharification and alcoholization activities of LBAW were obviously stronger than those of HBAW, and the variation trend of physicochemical parameters of the two kinds of *Hongqu* rice wine (HBAW and LBAW) is similar to the results described by a previous study (Liu et al., 2020).

### 3.2 The dynamics of biogenic amines

**Fig. 2:**
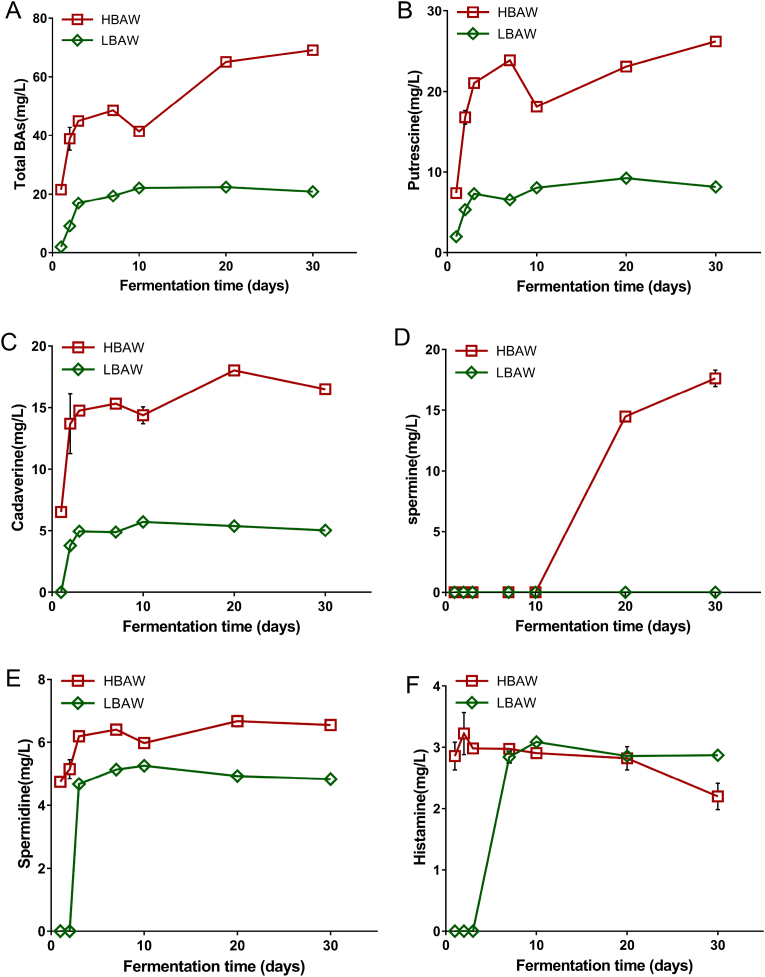
Dynamic changes of total biogenic amines (A), putrescine (B), cadaverine (C), spermidine (D), spermidine (E) and histamine (F) during the traditional brewing of two types of *Hongqu* rice wine (LBAW and HBAW).

BAs are commonly found in traditional fermented foods, and are closely related to human health. Appropriate BAs are beneficial to human health, while excessive intake of BAs will have toxic effects on human health (Zhang et al., 2019). As shown in Fig. 2A, the total biogenic amines (TBAs) contents of both LBAW and HBAW increased sharply at the initial stage of fermentation, and then tended to increase slowly, but the TBAs level of HBAW was significantly higher than that of LBAW. It is worth noting that on the 10th day of fermentation, it was observed that the BAs content of HBAW decreased slightly, which may be due to the high alcohol content of HBAW on the 7th day of fermentation, which may have an inhibitory effect on some BAs producing microorganisms, resulting in a decrease of BAs content in rice wine. According to a previous research report, a similar phenomenon was also observed during the fermentation of Shaoxing rice wine (Xia et al., 2018). Specifically, in the brewing process, the dominant BAs in HBAW include putrescine, cadaverine, spermine, spermidine and histamine, while, the main BAs in LBAW are composed of putrescine, cadaverine, spermidine and histamine.The levels of putrescine, cadaverine, spermine and spermidine were higher in HBAW than in LBAW (Fig. 2B–E). Spermine was not detected in LBAW, but a high level of spermine was detected in HBAW after 10 days of fermentation, and its content reached 17.62 mg/L on the 30th day of fermentation (Fig. 2D). In addition, there was a certain amount of histamine in HBAW during the whole fermentation process. However, the emergence of histamine in LBAW was observed on the 3rd day of fermentation and reached almost the same level as HBAW (Fig. 2F).

### 3.3 The dynamics of volatile organic compounds

**Fig. 3:**
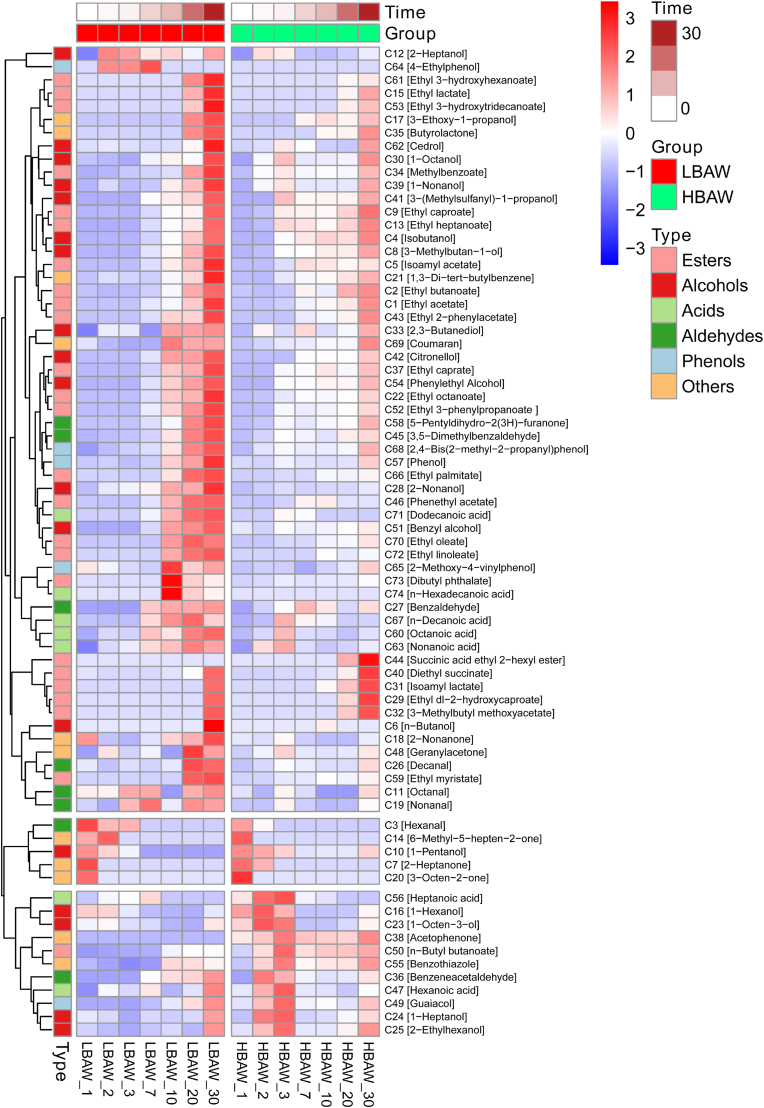
Heat map analysis of the dynamic changes in volatile flavor components during the traditional brewing of two types of *Hongqu* rice wine (LBAW and HBAW).

The characteristics of volatile organic compounds (VOCs) are the key indicators to evaluate the flavor quality and fermentation status of rice wine. As shown in Fig. 3, it was found that the characteristics of VOCs of LBAW and HBAW were obviously different through heatmap analysis of the abundance of VOCs in brewing process, the diversity and contents of VOCs of LBAW were significantly higher than those of HBAW. The profile of VOCs is one of the important factors contributing to the flavor quality *Hongqu* rice wine. High-quality *Hongqu* rice wine generally has a variety of VOCs, so it has a rich and harmonious aroma. In addition, hexanal [C3], 6-methyl-5-hepten-2-one [C14], 1-pentanol [C10], 2-heptanone [C7], 3-octen-2-one [C20] and 1-hexanol [C16] wre abundant in the early stage of LBAW and HBAW fermentation, and then gradually decreased in the rest stages. Among them, hexanal has green grassy aroma (Chen et al., 2013). Hexanal, ethyl hexanoate, benzaldehyde have been found to be key volatile aroma compounds of Shaoxing rice wine (Yu et al., 2019). However, in this study, high level of hexanal was detected in the early stage but was scarce in the late stage. Therefore, it is not considered as a core aromatic substance of *Hongqu* rice wine. In a previous study, Liu et al. found that VOCs such as 1-hexanol and ethyl isobutyrate were the main volatile flavor compounds in the early fermentation stage of *Hongqu* rice wine (Liu et al., 2020), which is consistent with the result of this study. Additionally, at the end of LBAW fermentation, more ethyl 3-hydroxyhexanoate [C61], ethyl lactate [C15], ethyl hydroxytridecanoate [C53], cedrol [C62], phenol [C57] are identified. While, succinic acid ethyl 2-hexyl ester [C44], diethyl succinate [C40], isoamyl lactate [C31], ethyl dl-2-hydroxycaproate [C29] and 3-methylbutyl methoxyacetate [C32] are abundant in the rice wine. It is well known that esters are the crucial VOCs contributing to the flavor characteristics of rice wine, involving fruity, flowery and sweet odors (Hu et al., 2018). In addition, diethyl succinate has sweet flavor characteristics and was proven to be a key aroma compound (Yang et al., 2017). Moreover, ethyl 3-hydroxyhexanoate and palmitic acid were determined as the unique aromatic components of *Hongqu* rice wine in comparison with the VOCs of *Y*aoqu rice wine (Li et al., 2020).

**Fig. 4:**
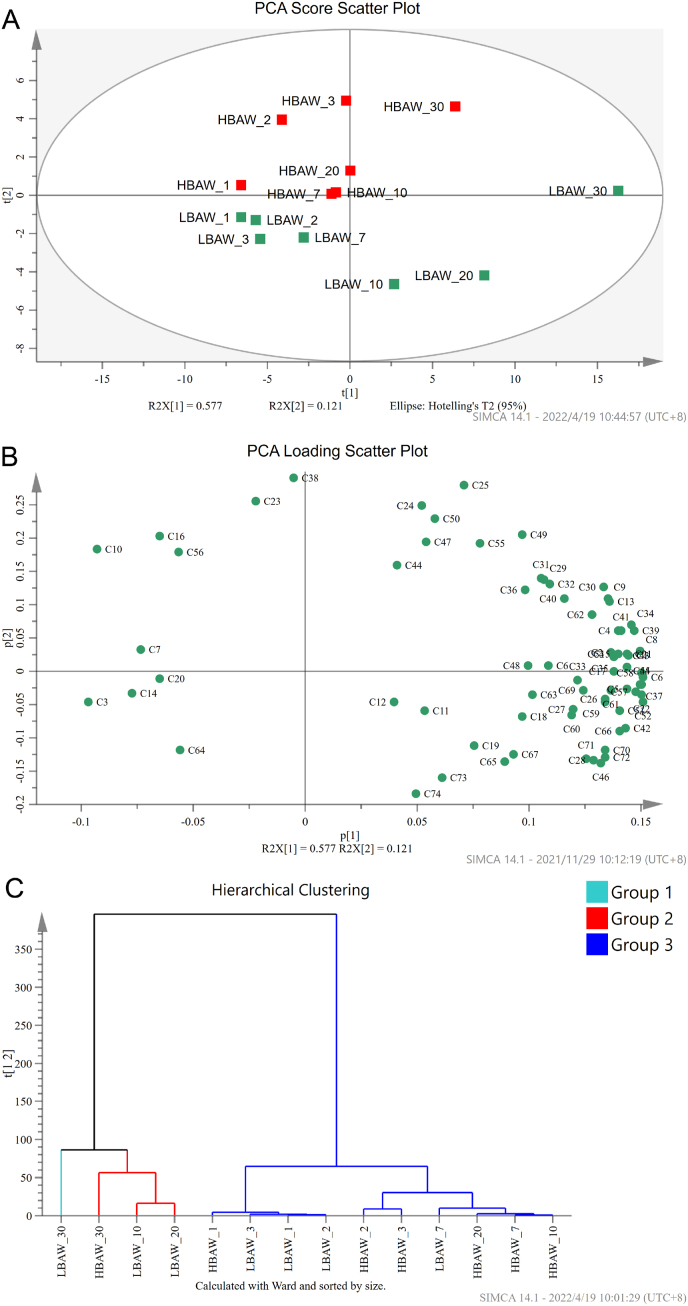
Principal component analysis (PCA) and Hierarchical clustering analysis (HCA) of volatile flavor components during the traditional brewing of two types of *Hongqu* rice wine (LBAW and HBAW). (A) PCA score plot. (B) PCA loading plot. (C) Hierarchical clustering diagram.

**Fig. 5:**
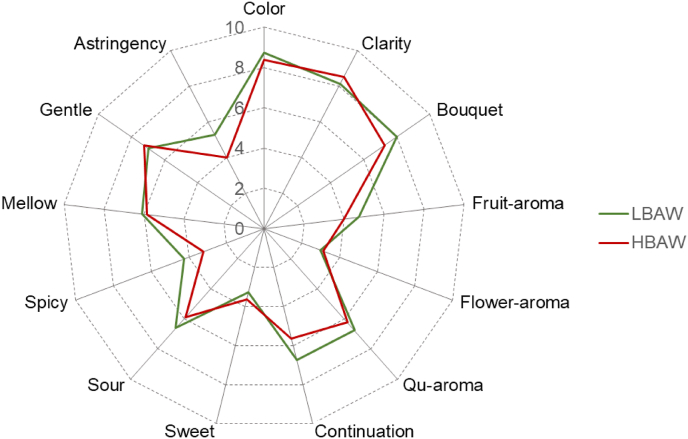
Sensory profiles of the two types of *Hongqu* rice wine (LBAW and HBAW) with the ratings given by trained panel.

The principal component analysis (PCA) of VOCs in the brewing process of both LBAW and HBAW was carried out. As shown in Fig. 4A, the VOCs profiles of the two kinds of *Hongqu* rice wine (HBAW and LBAW) are obviously different. Among them, the VOCs of HBAW are mainly concentrated in the first and second quadrants, while the VOCs of LBAW are mainly concentrated in the third and fourth quadrants of PCA score scatter plot. According to PCA loading scatter plot, it can be found that wine mash sample from HBAW_30 was mainly characterized by acetophenone [C38], n-butyl butanoate [C50], benzothiazole [C55]. Wine mash sample from LBAW_30 was mainly characterized by ethyl acetate [C1], isoamyl acetate [C5], 3-methylbutan-1-ol [C8], ethyl lactate [C15], 1,3-di-tert-butylbenzene [C21], ethyl octanoate [C22], ethyl caprate [C37], ethyl 2-phenylacetate [C43], 3,5-dimethylbenzaldehyde [C45], ethyl 3-phenylpropanoate [C52], ethyl 3-hydroxytridecanoate [C53], phenylethyl alcohol [C54], phenol [C57], 5-pentyldihydro-2-3(H)-furanone [C58] and 2,4-bis(2-methyl-2-propanyl) phenol [C68] (Fig. 4B). HBAW and LBAW have distinct flavor characteristics. Compared with HBAW, LBAW not only has higher safety due to lower BAs level, but also has more aromatic flavor substances. As shown in Fig. 5, LBAW was more prominent than HBAW in color, bouquet, fruit-aroma, continuation and mellow. Besides, the astringency and sour taste of LBAW were stronger than those of HBAW. Overall, the sensory evaluation showed that LBAW has better sensory quality than HBAW.

### 3.4 The dynamics of microbial communities

**Fig. 6:**
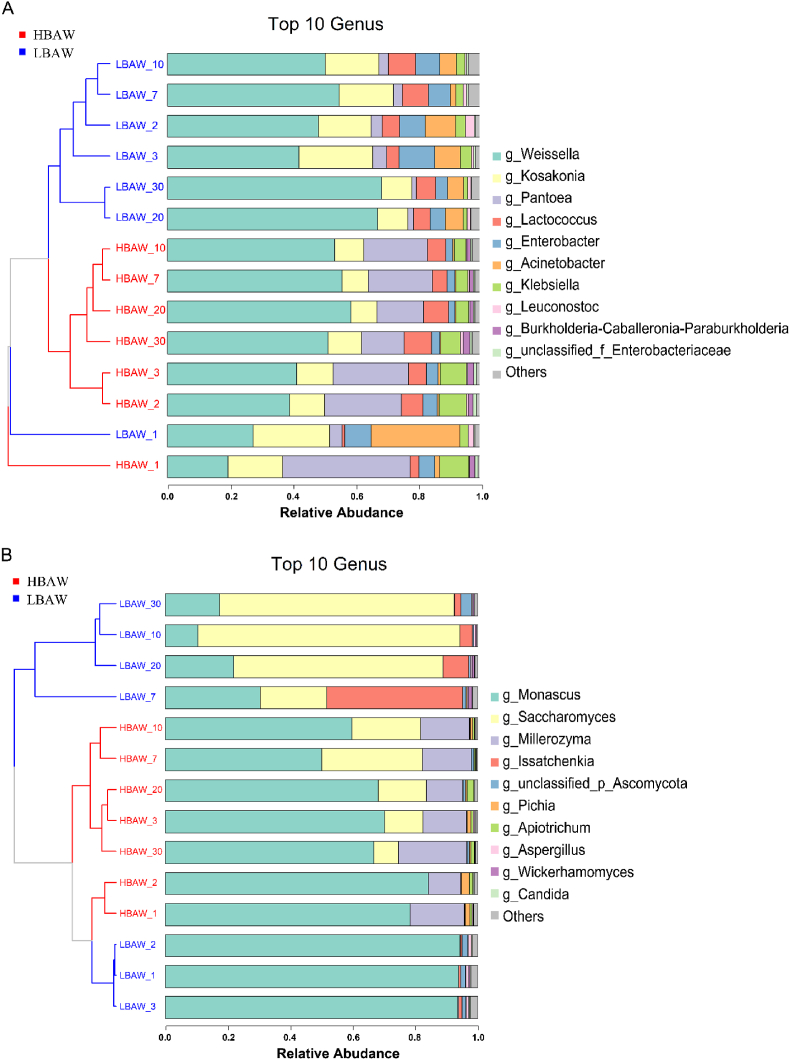
Stacked histogram of the relative abundance of the predominant microbial taxa at the genus level. (A) Bacterial taxa. (B) Fungal taxa.

The traditional brewing process of *Hongqu* rice wine is a process of “co-fermentation of multiple microbes”, which is involved a variety of microorganisms. As shown in Fig. 6, the stacked histogram of microbial community distribution showed that *Weissella*, *Kosakonia*, *Pantoea*, *Lactococcus*, *Enterobacter*, *Acinetobacter*, *Klebsiella*, *Leuconostoc*, *Burkholderia-Caballeronia-Paraburkholderia* and unclassified_f_*Enterobacteriaceae* were the main bacterial genera during the traditional fermentation of HBAW and LBAW. According to previous studies, *Burkholderia* is the predominant bacterial genus in *Hongqu* (Liu et al., 2018; Lv et al., 2013). In this study, it was found that *Burkholderia-Caballeronia-Paraburkholderia* was less abundant in LBAW, but more abundant in HBAW. It is worth noting that *Weissella* was dominant in the brewing process of both HBAW and LBAW. Meanwhile, the relative abundance of *Weissella* increased gradually with the progress of fermentation, and its relative abundance in HBAW was significantly lower than that in LBAW. In addition, at the early stage of brewing, abundant *Kosakonia* was found in both HBAW and LBAW, and its relative abundance in HBAW was lower than that in LBAW on the first day of brewing. Subsequently, the relative abundance of *Kosakonia* in LBAW decreased gradually, approaching 9.67% at the end of brewing (day 30). Meanwhile, the relative abundance of *Kosakonia* in HBAW is fluctuated and declined to 10.69% on the 30th day of fermentation. In addition, the relative abundance of *Pantoea*, *Enterobacter*, *Acinetobacter*, *Klebsiella* and unclassified *Enterobacteriaceae* displayed downward trend during the fermentation of both HBAW and LBAW. It is noteworthy that HBAW has more *Pantoea* (at least 5 times of LBAW in the relative abundance). It was known that *Pantoea* not only can produce yellow pigments, but also are the conditional pathogens (De Maayer et al., 2017).

From Fig. 6B, we can also observe the dynamic succession of fungal communities at the genus level during the brewing process of the two *Hongqu* rice wines. *Monascus*, *Saccharomyces*, *Millerozyma*, *Issatchenkia*, unclassified_p_*Ascomycota*, *Pichia*, *Apiotrichum*, *Aspergillus*, *Wickerhamomyces* and *Candida* are the main fungal genera in the wine making process. There are obvious differences in the structure of fungal flora between HBAW and LBAW. Specifically, *Monascus* is the predominant microorganism in HBAW brewing process, and its relative abundance accounts for more than 50% of the total fungal community. Moreover, the relative abundance of *Millerozyma* displayed a cosine increasing trend. In addition, the relative abundance of *Saccharomyces* rised on the third day of fermentation and showed a spiral upward trend in the following fermentation. While, the relative abundance of *Monascus* was more than 90% in the early stage of LBAW fermentation (1st day to 3rd day), but it dramatically declined to 30.48% at the 7th day and 17.47% at the end of fermentation. *Saccharomyces* became one of the dominant fungi on the 7th day of fermentation, and then dominated the subsequent fermentation process. Previous study had shown that *Monascus* has excellent ability to produce liquefaction and and saccharifying enzymes (Zhou et al., 2021). In this study, high-throughput sequencing results showed that *Monascus* has a relatively higher abundance in HBAW fermentation than LBAW, so the reducing sugar content in HBAW was obviously higher than that of LBAW at the early stage of fermentation. However, due to the rapid propagation of *Saccharomyces* and *Weissella* in LBAW at the later stage of fermentation, the reducing sugar content in LBAW decreased significantly faster than that in HBAW. It is well known that *Saccharomyces* is one of the main microorganisms that consume reducing sugars to produce ethanol. In this study, we found that the relative abundance of *Saccharomyces* in LBAW was higher than that of HBAW, which was consistent with the above trend of ethanol content. Moreover, early in fermentation of LBAW, the relative abundance of *Issatchenkia* increased sharply to 43.44% on the 7th day and then reduced rapidly during the subsequent fermentation. It was also found that there were more *Millerozyma* and *Apiotrichum* in the brewing of HBAW than in LBAW. However, LBAW contained more *Issatchenkia* and *Wickerhamomyces* than HBAW, and almost no *Issatchenkia* and *Wickerhamomyces* were found in HBAW during the whole fermentation process.

### 3.5 Correlation analysis of microbiota and metabolites

**Fig. 7:**
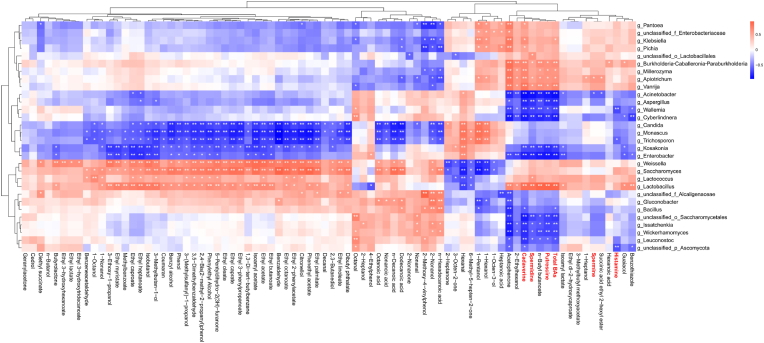
Correlation analysis between the volatile components/biogenic amines and the predominant microbial taxa at the genus level.

During the traditional fermentation of *Hongqu* rice wine, the VOCs and microbiota compositions changed dynamically with the brewing process (Liu et al., 2020). Microorganisms proliferate in the brewing process and use the winemaking substrate (glutinous rice) for growth and nutrient metabolism to produce a variety of metabolites favorable to flavor characteristics. Therefore, microbiota plays an important role in the formation of complex flavor components of rice wine. As shown in Fig. 7, Spearman correlation analysis was performed to analyze the association between the key microbial genera with metabolites in the brewing process of *Hongqu* rice wine (HBAW and LBAW), and it was found that some key microbial genera were highly correlated with volatile flavor components and biogenic amines. Specifically, spermidine, cadaverine, putrescine and total BAs were negatively correlated with *Acinetobacter*, *Aspergillus*, *Wallemia*, *Cyberlindnera*, *Kosakonia*, *Enterobacter*, unclassified_o_*Saccharomycetales*, *Issatchenkia*, *Wickerhamomyces* and *Leuconostoc*, but positively correlated with *Lactobacillus*, *Vanrija*, *Apiotrichum*, *Millerozyma*, *Burkholderia-Caballeronia-Paraburkholderia*. Based on previous studies, substantial evidences confirmed that a variety of lactic acid bacteria (LAB), including *Streptococcus*, *Lactococcus* and *Weissella* are the main contributors to BAs during rice wine fermentation (Luo et al., 2020; Xia et al., 2016; Liu et al., 2016). However, some LAB displayed the ability of inhibiting BAs biosynthesis during the food fermentation. For instance, Tabanelli et al. found that bacteriocinogenic Lactococcus lactis subsp. Lactis strains can inhibit the growth and metabolism of biogenic amine former LAB through producing *Streptococcal* peptide Z and nisin 481 (Tabanelli et al., 2014). Lee et al. found that *Lactobacillus brevis* degrades biogenic amines due to the expression of polycopper oxidase gene associated with polyamine oxidase (Lee et al., 2021). Moreover, it is noteworthy that only a few microbial genera have link with spermine, for instance, *Acinetobacter* has a negative correlation with spermine, and *Apiotrichum* displays positive relationship with spermine. Also, only unclassified_p_*Ascomycota*, *Trichosporon*, and *Wallemia* was revealed a significantly negative connection with histamine.

The volatile organic compounds are mainly derived from the brewing raw materials and microbial metabolites during the brewing and the aging process (Chen et al., 2020; Tian et al., 2022; Xu et al., 2018). Therefore, the microbial communities are also closely related to the flavor characteristics of rice wine (Jin et al., 2017). As showed in Fig. 7, the VOCs are strongly correlated with microbiota, for example, *Lactobacillus*, *Saccharomyces* and *Weissella* are positively correlated with VOCs, abundant in LBAW, including ethyl acetate, isoamyl acetate, 3-methylbutan-1-ol, ethyl lactate, 1,3-di-tert-butylbenzene, ethyl octanoate, ethyl caprate, ethyl 2-phenylacetate, 3,5-dimethylbenzaldehyde, ethyl 3-phenylpropanoate, ethyl 3-hydroxytridecanoate, phenylethyl alcohol, phenol, 5-pentyldihydro-2-3(H)-furanone and 2,4-bis(2-methyl-2-propanyl)phenol, the results is consistent with previous study (Huang et al., 2019); and those volatile components were negatively correlated with the relative abundance of *Candida*, *Monascus*, *Trichosporon*, *Kosakonia* and *Enterobacter*. In addition, *Weissella* and *Bacillus* showed significant positive correlation with most organic acids such as octanoic acid and nonanoic acid. A previous study also pointed out that *Bacillus* is a bacterium that can metabolize organic acids (Wang et al., 2014). Furthermore, succinic acid ethyl 2-hexyl ester, diethyl succinate, isoamyl lactate, ethyl dl-2-hydroxycaproate and 3-methylbutyl methoxyacetate were identified as the main flavor substances of HBAW. Succinic acid ethyl 2-hexyl ester showed significant negative correlation with *Acinetobacter* and but positive correlation with *Apiotrichum*. Meanwhile, isoamyl lactate was positively correlated with *Lactobacillus*, but negatively correlated with *Acinetobacter*, *Kosakonia* and *Enterobacter*. Previous study also indicated that *Lactococcus lactis*, *Burkholderia gladioli*, *Cronter helveticus*, *Wickerhamomyces anomalus*, *Saccharomyces cerevisiae*, *Aspergillus vitricola*, *Aspergillus penicillioides* and *Monascus purpureus* were closely related to the formation of a variety of volatile flavor substances (Liu et al., 2020).

### 3.6 Prediction of the key enzymes related to biogenic amines metabolism

**Fig. 8:**
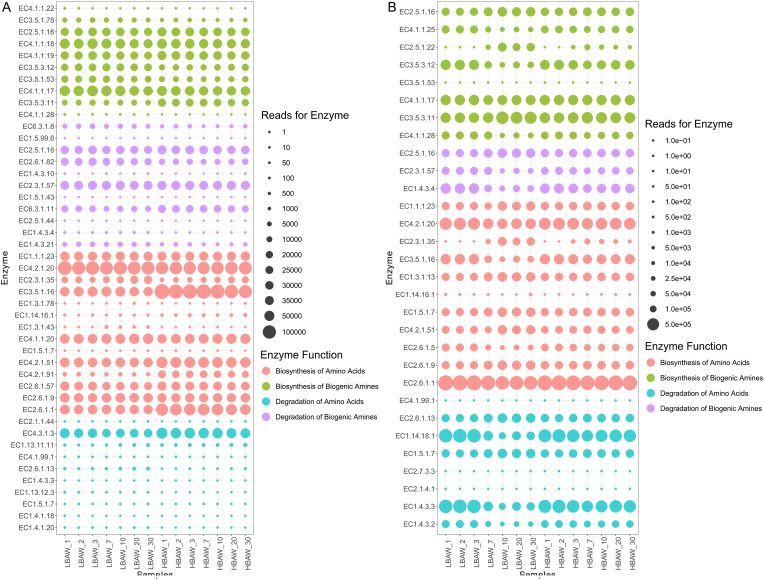
Bubble chart of dynamic changes in the abundance of the enzymes closely related to the synthesis and decomposition of biogenic amines during the traditional brewing of *Hongqu* rice wine (LBAW and HBAW). (A) Bacterial community; (B) Fungal community.

The temporal functional profiles for the metabolism of bacterial and fungal communities of *Hongqu* rice wine during fermentation were predicted by PICRUSt2 (Fig. 8A and B). In this study, we focused on the key enzymes involved in BAs metabolism, including the biosynthesis of amino acids and biogenic amine, the degradation of amino acids and biogenic amine. As the precursors of BAs, the synthesis and degradation efficiency of amino acids may affect the accumulation of BAs in *Hongqu* rice wine. The decarboxylation reaction of precursor amino acids is the main factor leading to the massive accumulation of BAs in wine (Ancin-Azpilicueta et al., 2008). The contents of putrescine, cadaverine and spermine in HBAW were significantly higher than those in LBAW. As can be seen from Fig. 8A, the abundance of the key enzymes in amino acid biosynthesis was obviously different in two types of *Hongqu* rice wine (LBAW and HBAW). The bacterial flora in HBAW produced more enzymes related to amino acid synthesis, especially acetylornithine deacetylase [EC 3.5.1.16, catalyzing the production of ornithine (Dou et al., 2011), a precursor of putrescine], arogenate dehydratase [EC 4.2.1.91, catalyzing the production of phenylalanine (Bross et al., 2017)], prephenate dehydratase [EC 4.2.1.51, catalyzing the decarboxylation of prephenate to phenylpyruvate (Werner et al., 2021)] and aspartate transaminase [EC 2.6.1.1, catalyzing the production of phenylalanine, tyrosine and glycine, glycine to produce putrescine]. The abundance of histidine ammonia-lyase [EC 4.3.1.3, catalyzing the degradation of histidine (Muramatsu et al., 2021)] was higher than that of LBAW, which was related to the synthesis of histamine. In addition, agmatinase [EC 3.5.3.11, catalyzing the formation of putrescine (Perez-Mozqueda et al., 2021)], arginine decarboxylase [EC 4.1.1.19, catalyzeing the formation of putrescine (Thongbhubate et al., 2021)] and spermidine synthase [EC 2.5.1.16, catalyzeing the formation of spermidine (Zou et al., 2021)] for the biosynthesis of biogenic amines were more abundant in HBAW than those in LBAW. A previous study based on PICRUSt showed that *Citrobacter*, *Acinetobacter*, *Lactobacillus*, *Exiguobacterium*, *Bacillus*, *Pseudomonas* and *Enterobacter* are the related microorganisms that can produce BAs during the traditional brewing of Chinese rice wine (Luo et al., 2020). They can express a series of amino acid decarboxylase genes and play an important role in the generation of BAs in Chinese rice wine (Luo et al., 2020).

## 4 Conclusion

This study investigated the formation of biogenic amines and volatile flavor compounds during the traditional brewing of *Hongqu* rice wine, and revealed their correlations with the microbial communities. We found that the total contents of biogenic amines in HBAW was higher than those in LBAW. Meanwhile, the volatile flavor characteristics of LBAW are more prominent than those of HBAW, and the sensory quality of LBAW is better. The contents of isoamyl acetate, ethyl lactate, ethyl caprate and phenylethyl alcohol were obviously higher in LBAW than those in HBAW. *Weissella*, *Kosakonia*, *Pantoea*, *Monascus*, *Saccharomyces*, *Issatchenkia* and *Millerozyma* were revealed as the predominant microbial genera in the traditional brewing of *Hongqu* rice wine. Spearman correlation analysis was performed to analyze the association between the key microbial genera with metabolites of *Hongqu* rice wine, and it was found that BAs were negatively correlated with unclassified_o_*Saccharomycetales*, *Cyberlindnera*, *Zygoascus*, *Aspergillus*, *Acinetobacter*, but positively correlated with *Lactobacillus*, *Pediococcus*, *Millerozyma* and *Apiotrichum. Lactobacillus*, *Pediococcus* and *Saccharomyces* were positively correlated with most of the volatile flavor components, while *Candida*, *Trichosporon* and *Monascus* were negatively correlated with most of the volatile flavor components. In addition, bioinformatical analysis based on PICRUSt demonstrated that the key microbial enzymes for BAs biosynthesis were more abundant in HBAW than those in LBAW. This study provides theoretical basis and technical support for improving the safety and flavor quality of *Hongqu* rice wine, and laid a solid scientific foundation for the healthy and sustainable development of *Hongqu* rice wine industry. In future research, metagenomics, metatranscriptomics and metaproteomics are necessary to clarify the formation and regulatory mechanism of BAs. Besides, modern technologies such as functional microbial enhancement, physical adsorption subtraction and exogenous additives are also necessary to regulate BAs formation in the traditional brewing of *Hongqu* rice wine.

## CRediT authorship contribution statement

**Gui-Mei Chen:** Methodology, Investigation, Writing – original draft, Validation. **Wen-Long Li:** Software, Investigation, Writing – original draft. **Shan-Gong Tong:** Investigation, Software. **Yun-Tao Qiu:** Methodology, Investigation. **Jin-Zhi Han:** Software, Investigation, Writing – original draft, Writing – review & editing. **Xu-Cong Lv:** Funding acquisition, Writing – review & editing, Supervision, Conceptualization, Project administration. **Lian-Zhong Ai:** Supervision, Conceptualization. **Jin-Yuan Sun:** Data curation, Writing – review & editing. **Bao-Guo Sun:** Supervision, Conceptualization. **Li Ni:** Supervision, Conceptualization, Writing – review & editing.

## Declaration of competing interest

The authors declare that they have no known competing financial interests or personal relationships that could have appeared to influence the work reported in this paper.
